# Human tumour vessel heterogeneity in ovarian cancer and its association with response to neoadjuvant chemotherapy

**DOI:** 10.1002/ctm2.1633

**Published:** 2024-04-15

**Authors:** Emmanuel M. Gabriel, Kulkaew Sukniam, Kyle Popp, Gabrielle Kowkabany, Harsheen K. Manaise, Kristopher Attwood, Anthony George, Qihui Zhai, Sanjay P. Bagaria, Keith L. Knutson, Joseph J. Skitzki, Matthew W. Robertson, Tri A. Dinh

**Affiliations:** ^1^ Department of General Surgery Division of Surgical Oncology Mayo Clinic Jacksonville Florida USA; ^2^ Department of Surgery Philadelphia College of Osteopathic Medicine Suwanee Georgia USA; ^3^ Department of Biology Florida State University Tallahassee Florida USA; ^4^ Department of Chemical Engineering University of Alabama Tuscaloosa Alabama USA; ^5^ Department of Medicine Government Medical College and Hospital Chandigarh India; ^6^ Department of Biostatistics Roswell Park Comprehensive Cancer Center Buffalo New York USA; ^7^ Department of Pathology Mayo Clinic Jacksonville Florida USA; ^8^ Department of Immunology Mayo Clinic Jacksonville Florida USA; ^9^ Department of Surgical Oncology Roswell Park Comprehensive Cancer Center Buffalo New York USA; ^10^ Department of Gynecological Oncology Mayo Clinic Jacksonville Florida USA

Dear Editor,

Aberrancies in tumour‐associated vasculature are known to negatively impact cancer‐related outcomes in a wide variety of cancers by creating barriers to drug delivery, which in turn limits drug efficacy.^1^, ^2^, ^3^ Direct, real‐time observations of tumour vascular heterogeneity have almost exclusively been studied using animal models. In this report, for the first time, we demonstrated a significant correlation between (1) tumour vessel heterogeneity, as directly observed using human intravital microscopy (HIVM), and (2) clinical outcomes in human subjects with ovarian cancer. Our group has previously investigated tumour‐associated vasculature in patients with melanoma, sarcoma, and carcinomatosis.^4^, ^5^ When compared to normal, non‐tumour (control) tissues, HIVM observations in tumour‐bearing areas revealed that tumour vessels (1) had smaller average diameters, (2) had lower velocities, and (3) were comprised of a high proportion of non‐functional vessels (did not support blood flow). Others have investigated the importance of tumour‐associated vessels specifically in ovarian cancer, whereby tumour vessel density and stability during disease progression were associated with poorer survival outcomes.^6^ In our own earlier trials, we were unable to detect statistically significant differences in treatment‐related outcomes, including response to neoadjuvant chemotherapy (NCT) and disease‐specific survival (DSS), likely because our previous studies were underpowered. In our current report, however, we have addressed the limitation in statistical power by expanding the study population to include only subjects with ovarian cancer who underwent NCT and surgery.

Between 1 January 2018 and 30 June 2023, a total of 25 subjects were enrolled on our study (inclusion and exclusion criteria provided in Table S1). Subject and tumour‐specific characteristics are shown in Table 1. All patients had carcinomatosis, received a minimum of three cycles of NCT, and underwent cytoreductive surgery with hyperthermic intraperitoneal chemotherapy (CRS‐HIPEC) with cisplatin. Following NCT, two patients achieved a complete pathologic response (CPR), 42.1% (12/25) achieved a partial response (PR), 31.6% (7/25) had stable disease (SD), and 26.3% (4/25) had progressive disease (PD). The median follow‐up period was 20.2 months. The median DSS was 25.2 months (95% confidence interval [CI] 14.4, not reached). The 1‐year and 3‐year DSS rates were 0.91 (95% CI 0.68–0.98) and 0.36 (0.20–0.78), respectively.

**TABLE 1:** Patient, tumour, and treatment‐related variables.

Variable		Overall
	*N*	25 (100%)
Age	Mean/Standard deviation	64.0/11.0
	Median	63.0
Race	White	21 (84.0%)
	Non‐White	4 (16.0%)
Body mass index	Mean/Standard deviation	28.1/6.8
	Median/Minimum/Maximum	26.9/17.4/46.7
Smoking status	Never	21 (84.0%)
	Former/Current	4 (16.0%)
Diabetes	None	21 (84.0%)
	Type II	4 (16.0%)
Subtype	Serous	24 (96.0%)
	Clear Cell	1 (4.0%)
Grade	Low	4 (16.0%)
	High	21 (84.0%)
Recurrence	No (initial)	19 (76.0%)
	Yes	6 (24.0%)
Prior surgery	None	11 (44.0%)
	Yes	14 (56.0%)
Approach	Open	20 (80.0%)
	Robotic	4 (16.0%)
	Laparoscopic	1 (4.0%)
Peritoneal carcinomatosis index	Mean/Standard deviation	9.8/7.7
	Median	8.0
Completeness of cytoreduction	Mean/Standard deviation	0.2/0.4
	Median	0.0
Adjuvant chemotherapy	No	4 (16.0%)
	Yes	21 (84.0%)
Complications	None	22 (88.0%)
	Yes (infection)	3 (12.0%)

Our methods for intraoperative HIVM have previously been described.^4^ Similar to our previous trials, statistically significant differences between tumour and normal blood vessels were observed. Table 2 shows the comparison of HIVM observations between subjects who had achieved CPR/PR and those who had SD/PD after NCT. Three statistically significant correlations were identified that support the notion that tumour aberrancy and non‐functionality were associated with a worse response to NCT. Patients who were found to have SD/PD after NCT had, on average, a lower number and density of functional blood vessels and a higher number of non‐functional vessels. The density of non‐functional tumour vessels was higher among those with SD/PD, though this just missed statistical significance after Holm–Bonferroni adjustment (*p* = .004). With regard to DSS, there was a statistically significant association between the proportion of non‐functional vessels at control (non‐tumour) sites whereby a lower proportion of non‐functionality correlated with improved DSS (hazard ratio [HR] = 0.34, 95% CI 0.12–0.93, *p* = .03).

**TABLE 2:** Comparison of human intravital microscopy (HIVM) vessel characteristics between subjects who had a complete pathologic response (CPR) or partial response (PR) and those who had stable disease (SD) or progressive disease (PD). The Holm–Bonferroni method was used to adjust for 16 comparisons, resulting in a significant p‐value of 0.003125 (0.05/16). Highlighted rows indicate statistically significant comparisons.

		CPR/PR^*^ *n* = 14 (56.0%)	SD/PD^†^ *n* = 11 (44.0%)	
Variable		Mean (Standard deviation)	Mean (Standard deviation)	*p*‐Value
# Functional vessels (per observed area)	Control	18.6 (8.3)	24.8 (9.5)	.079
	Tumour	15.4 (6.3)	7.0 (4.6)	.0027
# Non‐functional vessels (per observed area)	Control	2.7 (2.3)	3.2 (3.0)	.73
	Tumour	6.8 (1.9)	16.6 (6.6)	< .001
Density of functional vessels	Control	2.7 (0.9)	2.7 (0.8)	.93
	Tumour	1.9 (1.1)	0.6 (0.3)	< .001
Density of non‐functional vessels	Control	0.4 (0.3)	0.3 (0.3)	.63
	Tumour	0.9 (0.5)	1.6 (0.6)	.004
% Non‐functional vessels	Control	36.8 (11.6)	30.4 (4.2)	.3
	Tumour	25.1 (9.7)	20.5 (8.4)	.50
Diameter of functional vessels (μm)	Control	13.7 (10.6)	16.8 (11.8)	.48
	Tumour	20.3 (5.6)	15.1 (4.7)	.029
Diameter of non‐functional vessels (μm)	Control	12.7 (10.4)	9.9 (7.8)	.48
	Tumour	32.3 (14.4)	22.2 (8.6)	.19
Velocity of functional vessels (μm/s)	Control	294.3 (135.7)	356.1 (130.3)	.20
	Tumour	97.0 (39.6)	99.5 (40.2)	.87

* CPR = complete pathologic response, PR = partial response (per RECIST).

^†^ SD = stable disease, PD = progressive disease.

Pathologic tumour vessel measurements are closely correlated with HIVM measurements. Table 3 shows the Spearman correlation coefficients between pathologic and HIVM vessel densities or diameters. There was a statistically significant correlation between pathologic vessel density and HIVM vessel density at tumour areas for functional vessels. Similarly, there was a statistically significant correlation between pathological vessel diameter and HIVM‐measured diameters among functional blood vessels within tumour‐observed areas. Collectively, these data validated the HIVM vessel observations with gold‐standard pathologic analysis. Figure 1 illustrates representative pathologic slides with hematoxylin and eosin from subjects who obtained CPR/PR outcomes as well as from those with SD/PD. As expected, no gross or microscopic tumour was identified among subjects with CPR. Less residual tumour was present among samples obtained from subjects who attained PR compared to SD/PD. In addition, higher vessel density and larger blood vessels were identified for CPR/PR compared to SD/PD.

**TABLE 3:** Comparisons of (1) pathologic tumour vessel density (*n* = 23) with human intravital microscopy (HIVM) vessel density and (2) pathologic tumour vessel diameter (*n* = 23) with HIVM vessel diameter. The Holm–Bonferroni method was used to adjust for 10 comparisons, resulting in a significant *p*‐value of .005 (0.05/10). Highlighted cells indicate statistically significant correlations.

Spearman correlation coefficient *p*‐value				
	Density	Density	Density	Density
	HIVM tumour vessels	HIVM tumour vessels	HIVM control vessels	HIVM control vessels
	*Functional*	*Non‐Functional*	*Functional*	*Non‐Functional*
Density	0.72	0.47	0.21	0.30
*Pathologic vessels*	0.0001	0.026	0.34	0.16
Density		0.32	0.022	0.003
HIVM tumour vessels *Functional*		0.12	0.91	0.98
Density			0.60	0.31
HIVM tumour vessels			0.0021	0.14
*Non‐Functional*				
Density				0.16
HIVM control vessels *Functional*				0.41

	**Diameter**	**Diameter**	**Diameter**	**Diameter**
	**HIVM tumour vessels**	**HIVM tumour vessels**	**HIVM control vessels**	**HIVM control vessels**
	***Functional***	***Non‐functional***	***Functional***	***Non‐functional***
Diameter	0.57	0.19	0.10	0.38
*Pathologic vessels*	0.004	0.36	0.63	0.07
Diameter		0.02	0.13	0.17
HIVM tumour vessels		0.93	0.55	0.41
*Functional*				
Diameter			0.07	0.06
HIVM tumour vessels			0.76	0.75
*Non‐Functional*				
Diameter				0.14
HIVM control vessels				0.48
*Functional*				

**FIGURE 1:**
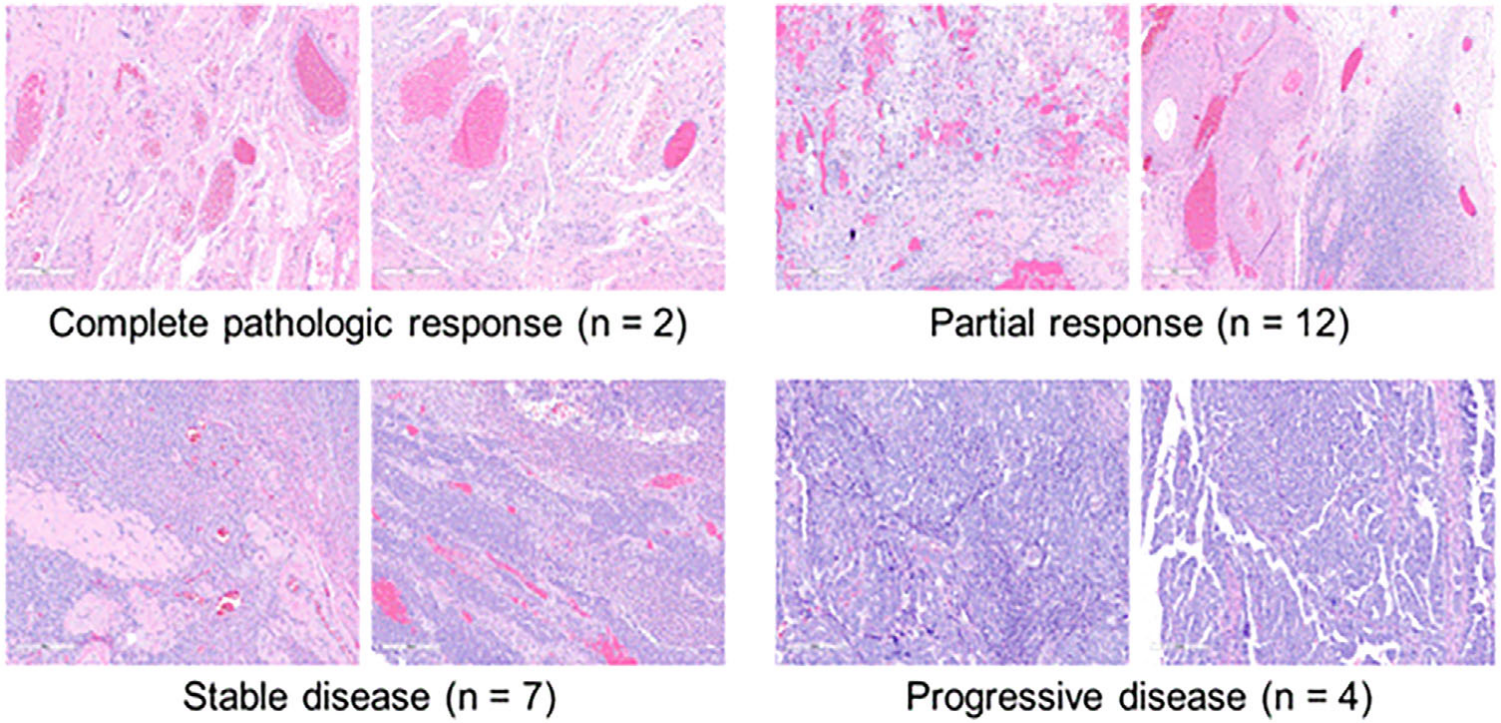
Pathologic hematoxylin and eosin (H&E) staining of representative samples of subjects with ovarian carcinomatosis with different responses to neoadjuvant chemotherapy (NCT). Samples from subjects who obtained complete pathologic response (CPR) or partial response (PR) had a higher density of blood vessels and larger blood vessel diameters, on average, compared to samples pertaining to stable disease (SD) or progressive disease (PD).

Our group has previously reported on a novel method to optimize tumour blood flow at the time of chemotherapeutic drug delivery.^5^ This approach was termed the dynamic control of tumour vessels and consisted of an appropriately timed bolus of saline and systemic phenylephrine. Our dynamic control protocol increased blood flow to the tumour bed as directly observed in real‐time by animal IVM and also improved anti‐tumour responses.^5^ While our technique has been employed only in animal models thus far, we anticipate bringing this to human trials now that HIVM has been increasingly utilized and the correlation between tumour vessel heterogeneity and oncologic outcomes has been further validated in this clinical trial. Future applications of HIVM are eagerly anticipated because the technology has the capacity to investigate drug delivery in ways that other imaging modalities cannot.

In conclusion, this is the first time that tumour vessel heterogeneity as observed by real‐time HIVM has been shown to correlate with response to systemic treatment and represents a significant advance from our prior clinical trials. While this association has been described in many animal models, this most recent human clinical trial has now validated a multitude of preclinical experiments and clinical studies that have demonstrated worse clinical outcomes associated with increased tumour vessel density and heterogeneity. Our pathologic correlates are critically important because they support the notion that tumour vessel heterogeneity at the surface of the tumour (observed via HIVM) was similar to that found within the more central tumour areas. Altogether, our findings in this trial are highly significant because these observed human tumour vessel aberrations directly affect blood flow and thus may significantly impact drug delivery. Overcoming tumour vessel structure and function as limitations to drug delivery is a target of many investigations.^7^, ^8^, ^9^, ^10^ Now, our trial provides real‐time evidence that abnormal HIVM tumour vessel characteristics correlate with treatment‐related outcomes, which has important implications for systemically administered cancer therapies. We anticipate utilizing HIVM for the direct monitoring of fluorescently labelled chemotherapeutic or targeted anti‐cancer agents, which is the focus of our future clinical studies.

## AUTHOR CONTRIBUTIONS

Drs. Gabriel, Bagaria, Knutson, Skitzki, Robertson and Dinh contributed to the study conception and design. Drs. Gabriel, Bagaria, Robertson and Dinh acquired the HIVM data. Drs. Gabriel, Sukniam, Popp, Kowkabany and Manaise analyzed the data. Dr. Attwood and Mr. George performed the statistical analysis. Dr. Zhai organized the pathologic analysis. Drs. Gabriel and Attwood, and Mr. George drafted the manuscript. All authors contributed to the revision, and each author gave final approval of the manuscript.

## CONFLICT OF INTEREST STATEMENT

The authors declare no conflict of interest.

## ETHICS STATEMENT

Our study was reviewed and approved by the Mayo Clinic Institutional Review Board.

## Supporting information

TABLE S1 Inclusion and exclusion criteria.

## Data Availability

Data will be made available upon request. Video data are in .mkt format, which is compatible with the HIVM microscope used in this clinical trial.
