# Supplementary material for: Human tumour vessel heterogeneity in ovarian cancer and its association with response to neoadjuvant chemotherapy
Source: Clin Transl Med. 2024 Apr 15;14(4):e1633. doi: 10.1002/ctm2.1633 (PMC11016937; doi:10.1002/ctm2.1633)
Supplement: Supplementary file 1 — TABLE S1 Inclusion and exclusion criteria. [file CTM2-14-e1633-s001.docx]

Supplementary Table 1. Inclusion and Exclusion Criteria

| **Inclusion Criteria** |
| --- |
| 1. Age ≥ 18 years of age. |
| 2. ECOG Performance Status of ≤ 2. |
| 3. Measurable tumor of ovarian etiology/histology on the peritoneal surface by direct visualization requiring surgical resection. |
| 4. Ovarian carcinomatosis that meets indications for cytoreductive surgery with hyperthermic intraperitoneal chemotherapy (CRS-HIPEC). |
| 5. Subject or legal representative must understand the investigational nature of this study and sign an Institutional Review Board approved written informed consent. |
| 6. Subject must have a skin prick test pre-operatively (at the time of the preoperative visit and after signing the informed consent) to determine any sensitivity to fluorescein. |
| **Exclusion Criteria** |
| 1. Uncontrolled illness including (but not limited to) active infection, symptomatic CHF, unstable angina, severe psychiatric illness, or extreme social situations that do not permit participation. |
| 2. Renal dysfunction as defined as a GFR < 45. |
| 3. Liver dysfunction as defined by Child-Pugh score > 5 or LFT’s 1.5x above normal range. |
| 4. Known allergy to fluorescein or a positive skin prick test to fluorescein. |
| 5. Pregnant or nursing female subjects, determined preoperatively with a urine pregnancy test. |
| 6. Unwilling or unable to follow protocol requirements. |
| 7. Any condition that excludes CRS-HIPEC as the standard of care (e.g. high disease burden where alternative treatments like systemic chemotherapy would be preferred). |
